# Memory reconsolidation as a tool to endure encoding deficits in elderly

**DOI:** 10.1371/journal.pone.0237361

**Published:** 2020-08-07

**Authors:** Leonela M. Tassone, Facundo A. Urreta Benítez, Delfina Rochon, Paula B. Martínez, Matias Bonilla, Candela S. Leon, Carolina Muchnik, Patricia Solis, Nancy Medel, Silvia Kochen, Luis I. Brusco, Malen D. Moyano, Cecilia Forcato

**Affiliations:** 1 Laboratorio de Sueño y Memoria, Departamento de Ciencias de la vida, Instituto Tecnológico de Buenos Aires (ITBA), Buenos Aires, Argentina; 2 Consejo Nacional de Investigaciones Científicas y Técnicas (CONICET), Buenos Aires, Argentina; 3 Centro de Neuropsiquiatría y Neurología de la Conducta- CENECON, Facultad de Ciencias Médicas, Universidad de Buenos Aires, Buenos Aires, Argentina; 4 Unidad Ejecutora de Estudios de Neurociencias y Sistemas Complejos, CONICET, Hospital El Cruce “Néstor Kirchner”, Universidad Nacional Arturo Jauretche, Florencio Varela, Argentina; Nathan S Kline Institute, UNITED STATES

## Abstract

Normal aging involves changes in the ability to acquire, consolidate and recall new information. It has been recently proposed that the reconsolidation process is also affected in older adults. Reconsolidation is triggered after reminder presentation, allowing memories to be modified: they can be impaired, strengthened or changed in their content. In young adults it was previously shown that the presentation of repetitive reminders induces memory strengthening one day after reactivation and the presentation of at least one reminder increases memory persistence several days after reactivation. However, until now this process has remained elusive in older adults. We hypothesize that older adults need a stronger reminder to induce memory strengthening through the reconsolidation process than young adults. To test this, we perform a three-day experiment. On day 1, participants learned 15 sound-word associations, on day 2 they received no reminders (NR group), one reminder (R group) or two rounds of reactivations (Rx2 group). Finally, they were tested on day 7. We found that, contrary to our hypothesis, older adults show a memory improvement triggered by repeated labilization/reconsolidation processes to an equal extent than young adults. These results open new perspectives into the use of reconsolidation to improve daily acquired information and the development of therapeutic home used tools to produce memory enhancement in healthy older adults or those with cognitive decline.

## Introduction

Memories are dynamic and they change through time. One of the processes that allow this modification is reconsolidation. It is triggered by a memory cue associated with the original information (reminder) inducing destabilization of the trace (labilization) and it implies a restabilization period dependent on protein synthesis and gene expression [1, 2]. This process is not unique to humans, it is conserved across species [3–5] and it is involved in different kinds of memories [6–12]. After reminder presentation, memories can be modified: impaired, strengthened or updated in content [7, 8, 13–19]. Regarding memory strengthening, Forcato et al. [18] trained young adults to associate five pairs of nonsense syllables. On day 2, they received one, two or no reminders and they were tested on day 3. They observed memory strengthening on day 3 only when at least two reactivations were triggered 24 h before [18]. However, if subjects were tested on day 7 memory strengthening was also observed for the group that received only one reminder [19]. They also found that if they analyzed the type of error at testing on day 3, there was a reduction of the confusion type error (when participants mixed the letters of a syllable or they wrote a syllable that did not exist on the list) for the group that received two reactivations compared to the other two groups [18]. Furthermore, they observed a reduction in void errors (when participants did not give any answers) on day 7, for the groups that were reactivated with one or two reminders, compared to the no reactivation group [19]. Thus, repeated reconsolidation processes could enhance memory precision and also memory persistence.

Normal aging is accompanied by complains in the ability to acquire, consolidate and recall new information. Perception, processing speed, working memory and controlled attention are impaired in normal aging and negatively affect the way in which the new information is acquired [20–24]. Furthermore, memory reconsolidation has also been suggested to be affected as well [25–27].

Jones et al. [25] studied the effect of aging in memory reconsolidation in rats and humans using two analogous episodic tasks. On day 1, old and young participants learned a set of objects [28], whereas rats learned feeder locations in context A (Set 1). On day 2, the memory was reactivated in context A and participants learned a new set of objects (Set 2) in the same context (Context A—reminder condition), or they learned the new set in a new context (Context B- no reminder condition). On day 3, they were tested for both sets of objects. Under this paradigm, reconsolidation is revealed by an increase in intrusions from Set 2 into Set 1 recall in the group that received the reminder previously to Set 2 learning compared to the no reminder condition, a phenomenon known as updating [8]. In both species, older subjects displayed a different pattern of results than younger subjects. Thus, younger rats and humans in the reminder condition falsely recalled significantly more items from Set 2 objects than the no reminder condition. However, in aged rats there was no difference between conditions in the level of intrusions. Older humans in the no reminder condition made significantly more intrusions than those in the reminder condition. Follow-up control experiments in aged rats and humans suggested that intrusions reflected general interference, independent of context manipulations. They concluded that contextual reminders were not sufficient to trigger memory reconsolidation in aged rats or aged humans, unlike in younger individuals [25].

Recently, Sandrini et al. [29] studied whether repetitive transcranial magnetic stimulation (rTMS) in young adults over the right Lateral Prefrontal Cortex (PFC), a region involved in the reactivation of episodic memories [30] strengthened an episodic memory after contextual reactivation. For that, all participants learned a list of 20 object-words on day 1 (context A). On day 2, the “PFC-R” group received the contextual reminder cue (context A) and 10 min later the rTMS to the right Lateral PFC. The “PFC-NR” received the rTMS to the right Lateral PFC but with no memory reactivation (context B) and the “Vertex-R” group received the rTMS over the Vertex after memory reactivation (context A). On day 3 the memory was tested. They observed memory improvement only in the group that received the rTMS to the right Lateral PFC after memory reactivation. Furthermore, they found that in older adults anodal transcranial direct current stimulation (tDCS) over the left Dorsolateral Prefrontal Cortex (DLPFC) with or without the reminder strengthened the word list and reduced forgetting compared to sham stimulation [26]. Thus, unlike younger adults [29], the no reminder condition (room B) would have reactivated the memory to an equal extent to the reminder condition (context A) in older adults. It is important to highlight that unlike young adults [29], being in the same hospital might have been more salient to older adults than the distinction between the two experimental rooms, and the no reminder group would have been reminded of the learning session (Day 1) and performed the same as the reminder group suggesting that contextual cues may be encoded but may not be appropriately bound to the target event [26].

Regarding reconsolidation memory updating, St. Jacques et al. [27] trained young and older adults in a museum tour (Day 1). On Day 3, memory was reactivated by presenting digital photos of a subset of museum stops followed by the presentation of novel lure photos from an alternate tour version. On Day 5, they had to recognize whether they have visited during the museum tour or not the stops shown in the photos. They found that when memories were reactivated, there was an increase in subsequent true and false memories, compared with baseline, in young and older adults. However, older adults had a smaller increase than young, reaching 10% less overall gain for reactivated memories. Taking this finding into account, they concluded that the magnitude of memory reactivation effect is reduced in aging.

The discrepancy between the above results could be due to older adults’ deficits at processing the reminder. Jones et al. [25] as well as Sandrini et al. [26] used contextual reminders (room A) to trigger memory labilization and they found no significant differences between reactivated and no-reactivated groups in older adults. On the other hand, Jacques et al., [27] used specific cues to trigger the process observing differences between reactivated and no-reactivated older groups, however to a lesser extent than young participants. Considering that contextualization is impaired in older adults, it could be interfering the reactivation process [25]. Thus, a reminder that triggers memory labilization/re-stabilization in young adults could be not strong enough to trigger labilization in older adults. Thus, we hypothesize that older adults need more rounds of reminder presentations than young adults to produce memory strengthening at the same extent. Here, we study reconsolidation in young and older adults using a sound-word paradigm as a noninvasive technique to induce repetitive reactivations in order to improve and increase the persistence of the original declarative memory.

## Materials and methods

### Participants

Fifty-four healthy older adults (37 females and 17 males) and forty-four young adults (19 females and 25 males) volunteered for the study.

Young adults were graduate and undergraduate students from Buenos Aires, and their ages ranged between 18–40 years (M = 21.3, SEM = 0.8). They had no history of neuropsychiatric disorders, and they did not use drugs. Prior to being enrolled in the experiment, subjects signed a written informed consent.

Older adults’ ages ranged between 60–86 years (M = 70.4, SEM = 1.1). They had completed primary school, with a mean of 9.3, SEM = 0.5 years of education and had no history of previous neurological or psychiatric disorders and they did not have hearing problems. Prior to being enrolled in the experiment, subjects completed a brief neuropsychological screening, including Addenbrooke´s examination test and Ineco Frontal Screening test to verify the absence of cognitive deficits [31, 32]. They had performances over the established cut-off point of 68/100 (ACE-R) and 23/30 (IFS Test), a cut-off established for healthy individuals with less than 12 years of formal education, meaning no suspect of dementia or executive dysfunction.

Data from 13 older adults were excluded from the analysis because they did not reach the learning criteria of more than 40% (six words) of correct responses at training (4), and did not come to the testing session on day 7 (9). Data from 2 young adults were excluded because they did not reach the learning criteria of more than 40% of correct responses at training.

### Experimental groups

Young and older adults were randomly assigned to one of three conditions: “No reminder”, “Reactivation”, “Double Reactivation” groups (Table 1).

**Table 1:** Demographic characteristics and neuropsychological assessment.

	Older adults				Young adults	
Groups	Age	Education	ACE-R	IFS	Age	Education
**NR**	67.9±1.6	9.9 ±1.0	90.6±1.3	25.5±0.4	20.7±1.1	13.4±0.5
**R**	69.3±1.6	9.6±0.7	89.9±1.2	26.3±0.5	23.1±1.9	13.5±0.5
**Rx2**	74.4±2.0	8.9±1.0	89.1±1.1	25.6±0.2	20.1±1.0	13.1±0.5
**p- value**	0.03	0.75	0.65	0.19	0.31	0.86

Mean age and mean years of education for young and older adults, and mean scores at Addenbrooke´s examination test R (ACE R), INECO Frontal Screening (IFS), ± SEM for older adults. NR, stands for the no reminder group; R, for one reactivation, and Rx2, for two reactivations.

#### “No reminder” group (NR)

Participants were trained on day 1 and tested on day 7. They did not receive the reactivation treatment on day 2 thus they did not attend to the lab on that day. Young and older NR groups were formed by 14 subjects each.

#### “Reactivation” group (R)

They were trained on day 1, received one round of reminder presentation on day 2 and were tested on day 7. Young and older R groups were formed by 14 subjects each.

#### “Double Reactivation” group (Rx2)

They were trained on day 1, received two consecutive rounds of reactivations on day 2 and they were tested on day 7. Young and older Rx2 groups were formed by 14 and 13 subjects, respectively.

### Experimental design

**Fig 1:**
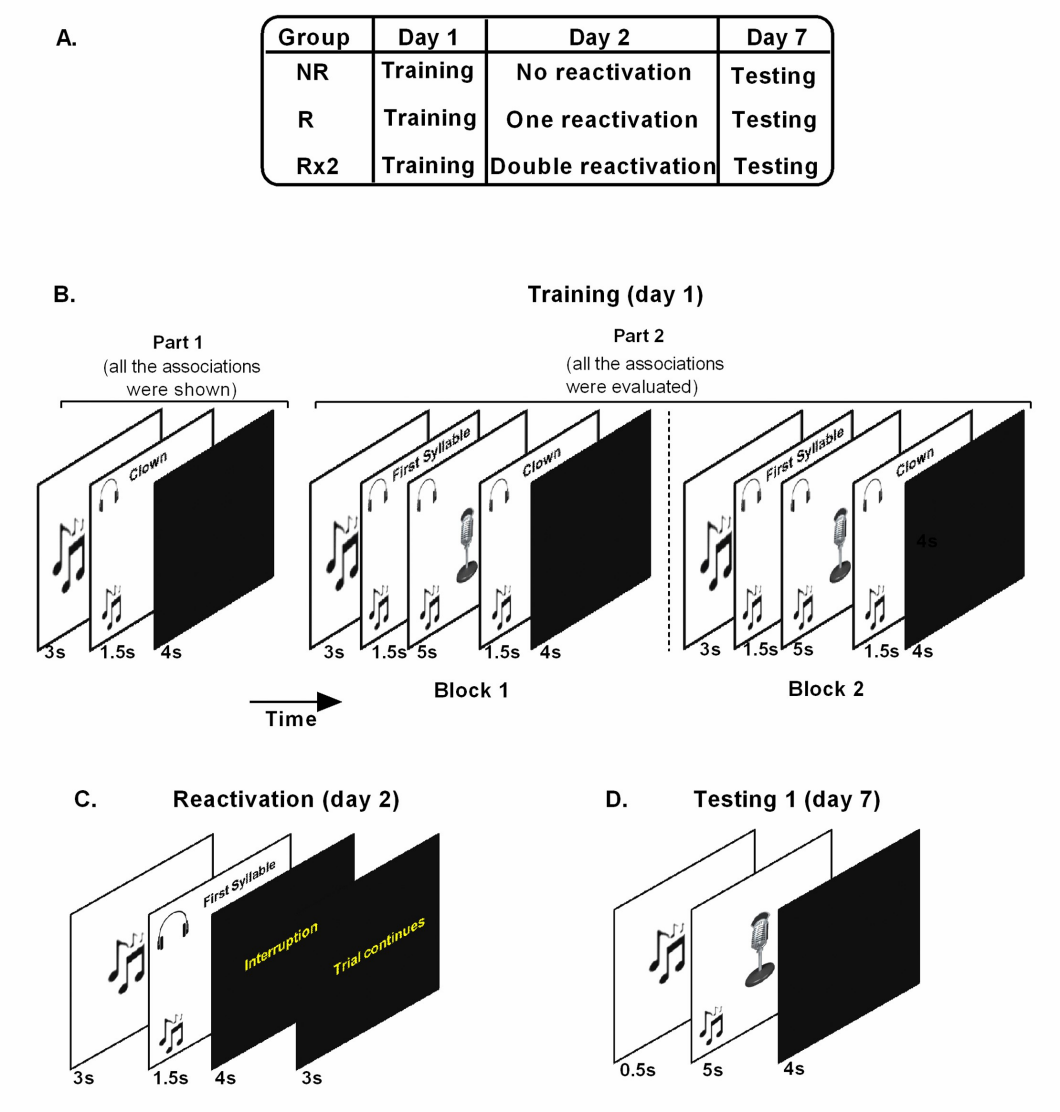
Experimental paradigm. (A) Experimental protocol. (B) Training (day 1) *Part 1*: The 15 sound-word associations were presented on the computer screen. The music image stands for the sound. The headphones image stands for the word presented in an auditory form. *Part 2*: The 15 associations were evaluated in two blocks. For each association, the sound and the first syllable of the associated word were presented and participants had to answer with the associated word aloud upon appearance of an image of a microphone on the screen. The correct answer was displayed written on the screen and via headphones. (C) Reactivation (day 2). Each sound was followed by the presentation of the first syllable of each word. Participants were instructed to answer with the associated word aloud every time the microphone appeared on the screen however, the microphone was never shown. This sequence was repeated until all associations were presented. (D) Testing (day 7). All the associations were tested. The sound was presented and participants had to answer with the associated word.

The study was performed in three days. Protocol and consent were approved by the Biomedical Research Ethics Committee of the Alberto C. Taquini Institute and the Hospital El Cruce Dr. Néstor Carlos Kirchner, in accordance to the principles expressed in the Declaration of Helsinki. On day 1, participants arrived at the lab between 9:00 to 11:00 h, they read and signed the written informed consent form. They performed the verbal fluency test and received the sound-word associations training. After that, they were allowed to leave the lab. On day 2, participants of NR group did not receive any treatment thus they did not have to assist to the lab that day. The other two groups arrived at the lab between 9:00 to 11:00 h. The R group received one round of reminder presentation while the Rx2 group received two rounds. After that, they completed a verbal fluency test. On day 7, all participants arrived at the lab between 11:00 to 13:00 h and were tested (Fig 1A). After that they completed the verbal fluency task. All participants performed the three sessions in the same experimental room.

#### Training (day 1)

The training included two parts (Fig 1B). In part 1, the 15 associations of sounds and Spanish words were presented on the computer screen. The words were nouns and each one had three syllables and six letters. The sound was semantically related to the word. Each sound was presented for 3 seconds followed by the associated word overlaid for 1.5 s. Each word was presented in an auditory and written form. After that, a black screen was shown for 4 s. The sequence was repeated until the 15 sound-word associations were shown. Immediately after, the part 2 started. The 15 associations were evaluated in two blocks to determine the level of training (each block was formed by the 15 associations randomly presented). The sound was presented alone for 3 s and remained for 1.5 s more overlaid by the first syllable of the associated word presented just once (e.g.: the sound of laughing children and the first syllable of the associated word *PAYASO “PA”*, clown). The sound continued for 5 s more while an image of a microphone appeared on the screen and participants had to complete orally with the associated word. The sound remained for 1.5 s more and the correct associated word was presented in an auditory and written form followed by a black screen which was shown for 4 s. The answers were recorded. The procedure took 10 min.

#### Reactivation (day 2)

In order to induce memory labilization, we used incomplete reminders. It has been shown that reminders formed by only parts of the original learning information, for example when an expected reinforcement after the presentation of the cue is omitted (e.g. single cue syllable), are more effective in triggering memory labilization reconsolidation than complete ones (reminders formed by the complete stimulus association [15].

Before reactivation participants were instructed to hear the sound and first syllable of the associated word and answer with the associated word each time they saw an image of a microphone on the screen. After the instruction, each sound was presented alone for 3 s and continued 1.5 s more overlaid by the first syllable of the associated word auditory presented once. After that, a message announcing that the trial was interrupted appeared on the screen for 4 s, followed by a message indicating that the trial continued (3 s). This sequence was repeated until the 15 associations were presented. Participants were instructed to pay attention to entire procedure, and to respond when the microphone appeared. However, the microphone was never shown during reactivation. The procedure took 3 min (Fig 1C).

#### Testing (day 7)

Participants were instructed to hear each sound and to answer with the associated word each time they saw the image of the microphone on the screen. After the instruction the sound was presented alone for 0.5 s and then continued for 5 s more overlaid with the image of the microphone of the screen. After that, a black screen was shown for 4 s. The procedure was repeated until the 15 associations were tested. No feedback was given and the answers were recorded. The procedure took 3 min (Fig 1D).

### Neuropsychological assessment

Older adults completed a brief neuropsychological screening at the Integral Medical Center before participating in the study, including Adenbrooke´s Cognitive Examination Test and INECO Frontal Screening Test (Table 1).

#### Addenbrooke´s cognitive examination R

ACE-R is a cognitive screening test that evaluates six domains: orientation, attention, memory, verbal fluency, language and visuospatial function. The memory item includes the evaluation of episodic and semantic memory. ACE-R is a useful tool to distinguish between the cognitive changes present in the normal aging process and the cognitive decline compatible with Alzheimer's dementia or other dementias. The administration takes 15 minutes, and the maximum score is 100, with a cut-off point of 68 for healthy individuals with less than 12 years of formal education [32].

#### Ineco frontal screening test

IFS Test is a brief screening tool for the detection of executive dysfunction, evaluating three groups of tasks: response inhibition and set shifting, capacity of abstraction, working memory, setting a cut-off point of 23/30, according to the years of formal education, the total score obtained is useful to discriminate healthy from demented subjects [31].

#### Verbal fluency task (control measure)

We also tested the verbal fluency at two different time points during the experiment (training on day 1, testing session on day 7). Verbal Fluency is the ability to evoke a spontaneously fluent speech. This test is useful to evaluate the semantic storage capacity, the ability to recover information and the indemnity of executive functions [33]. In the present study, subjects had to say, within one minute, all the words they could think about that begins with a specific letter. The letters were presented in the same order to all subjects no matter the group they were in (In day 1, the letter “P” was presented and in day 7 was the letter “C”).

### Statistical analysis

Statistical analysis was done with SPSS version 25 (IBM Corporation). We calculated the absolute memory change as the number of correct responses at testing on day 7 minus the number of correct responses at the second block of training on day 1. Thus, positive values mean memory gain and negative values, memory loss.

We further calculated the normalized memory change as (the number of correct responses at testing minus the number of correct responses at training) multiplied by 100% and divided the number of correct responses at training.

We analyzed the training session (second block of training), the testing session (day 7-S1 Table), the absolute memory change and the normalized memory change with separately two-way ANOVAs with type of reminder as between subjects’ factor with three levels (NR, R, Rx2) and age as between subjects factor with two levels (young, older), followed by Bonferroni Post-hoc tests. To determine if there was a significant decrease in memory from day 1 to 7, we further performed two tailed one sample t-test for each group compared to the value zero (no memory change).

For a deeper analysis, we classified the errors at testing into three different types. *Void errors*, when participants did not answer at all. *Confusion errors*, when participants answered with a word that did not belong to the list. *Intralist errors*, when participants incorrectly answered with another word of the list. We first determine for each subject the 100% of errors as the total number of errors done for each subject at testing and then we calculated the proportion of the 3 types of errors for each subject. For intralist and confusion type errors we had to transform the variable to Square root to accomplished ANOVA assumptions to reach normal distribution and homogeneity of variance. We performed a two-way ANOVA for the three type of errors (void, confusion and intralist), with type of reminder as between subject’s factor with three levels (NR, R, Rx2) and age as between subjects’ factor with two levels (young, older). Followed by Bonferroni Post-hoc tests. Alpha was set at 0.05.

In order to examine if reconsolidation process could be affected by other variables such as age or general cognitive function, we further analyzed the ACE, IFS, age and years of education and memory change in the three types of reminder condition (NR, R, Rx2) in older adults with Pearson correlations. Alpha was set at 0.05, we performed no corrections for multiple comparisons.

The fluency task and psychological tests were analyzed with one-way ANOVAs, with type of reminder as between subjects’ factor with three levels (NR, R, Rx2), followed by Bonferroni post-hoc tests.

## Results

In order to study memory strengthening in young and older adults trough the reconsolidation process, we ran a three-day experiment (Fig 1A). On day 1, participants learned the sound-word associations. On day 2, participants received one, two or no rounds of reactivation and they were finally tested on day 7 for the sound-word associations.

**Fig 2:**
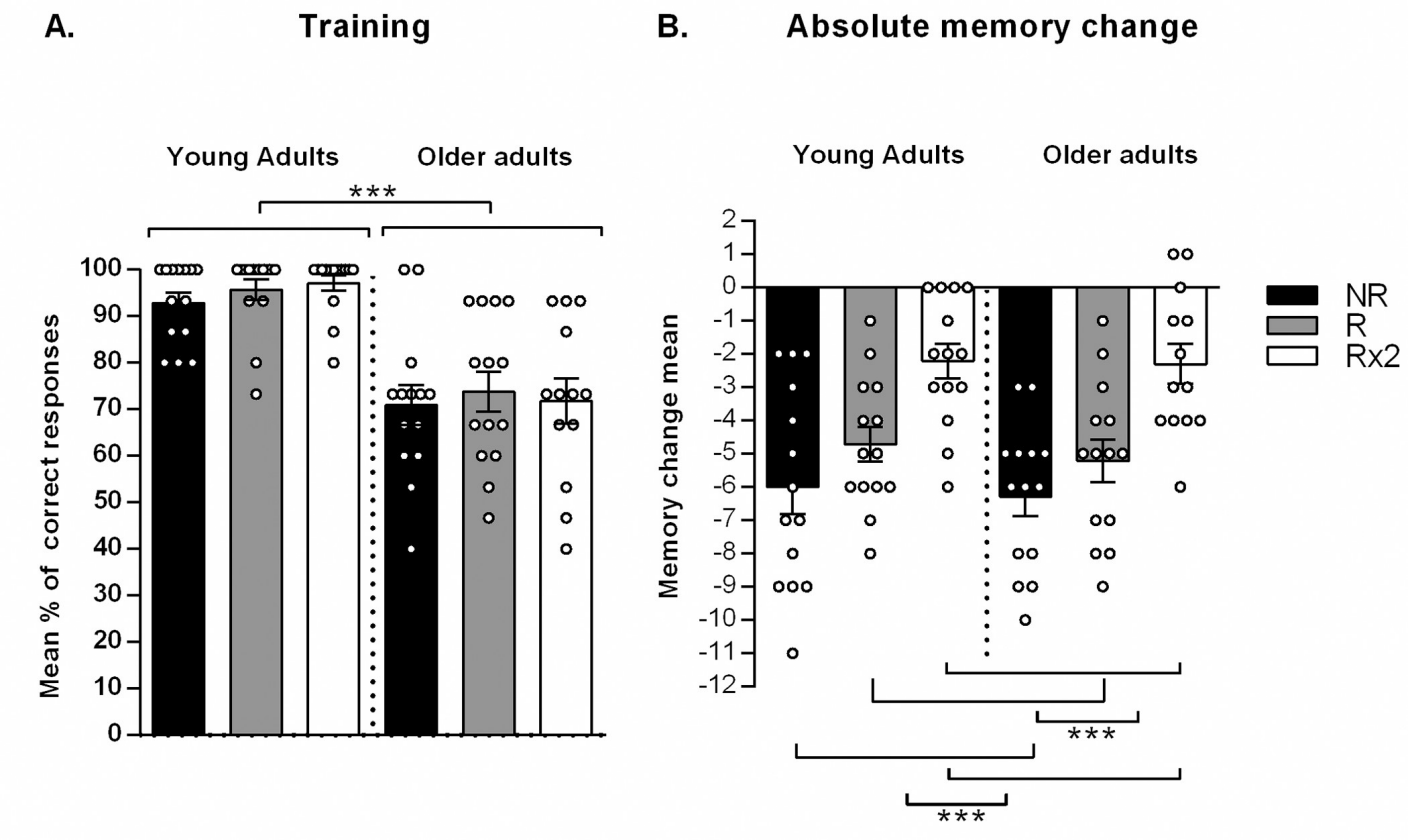
Reconsolidation memory strengthening. (A) Mean percentage of correct responses at training ± SEM for young and older adults. (B) Absolute memory change mean (correct responses at training minus correct responses at testing) ± SEM for young and older adults.

As it was previously reported, older adults had worse performance at training (Fig 2A, for older adults: NR: 71.0 ± 4.3, R: 73.8 ± 4.3, Rx2: 71.8 ± 4.8; For young adults: NR: 92.9 ± 2.3, R: 95.7 ± 2.3, Rx2: 97.1 ± 1.7; F_age_(1,77) = 66.751 p<0.001) probably due to the normal deficits at encoding information in aging [20, 33, 34]. Furthermore, there was no significant difference between type of reminder at training session (F_reminder_(2,77) = 0.417 p = 0.660) nor age x type of reminder interaction (F_agexreminder_(2,77) = 0.163 p = 0.850). To study the effect of memory reactivation we calculate the absolute memory change (correct responses at testing minus correct responses at training Fig 2B). There was no significant interaction between age and reminder condition (For young adults: Rx2:- 2.2 ± 0.5, R: -4.7 ± 0.5, NR: -6.0 ± 0.8. For older adults: -Rx2: -2.3 ± 0.6, R: -5.2 ± 0.6, NR: -6.23± 0.6; F_agexreminder_(2,77) = 0.056 p = 0.946) and memory change was similar between young and older adults (F_age_(1,77) = 0.279 p = 0.599). However, it was significantly different between reminder conditions (F_reminder_(2,77) = 19.817 p<0.001). Specifically, the Rx2 group showed better performance that the NR and R groups (both ps<0.001), but no differences were found between the NR and R groups (p = 0.208). Furthermore, all conditions showed significant decay between the number of correct responses on day 1 and day 7 (For young adults: T-test: NR T_13_ = -7.38 p<0.001, R T_13_ = -8.91 p<0.001 and Rx2 T_13_ = -4.29 p = 0.001; For older adults: T-test: NR T_13_ = -10.29 p<0.001, R T_13_ = -8.27 p<0.001 and Rx2 T_12_ = -3.82 p = 0.002) possibly due to forgetting, however, if two rounds of reminders were presented memory was more protected against forgetting.

We further analyzed memory change normalized to the level of training (Table 2). There was no significant interaction between age and reminder condition _(Fagexreminder_(2,77) = 0.493 p = 0.612). Older adults showed higher decay in normalized memory change (F_age_(1,77) = 7.511 p = 0.008). There were significantly differences between reminder conditions (F_reminder_(2,77) = 19.779 p<0.001). Specifically, the Rx2 group showed better performance that the NR and R groups (both ps<0.001), but no differences were found between the NR and R groups (p = 0.089).

**Table 2:** Normalized memory change.

Groups	Young adults	Older adults
**NR**	-43.8±5.9	-61.2±6.6
**R**	-33.9±4.5	-46.9±5.3
**Rx2**	-15.0±3.6	21.5±6.5

Normalized memory change ± SEM for young and older adults.

NR, stands for the no reminder group; R, for one reactivation, and Rx2, for two reactivations.

**Fig 3:**
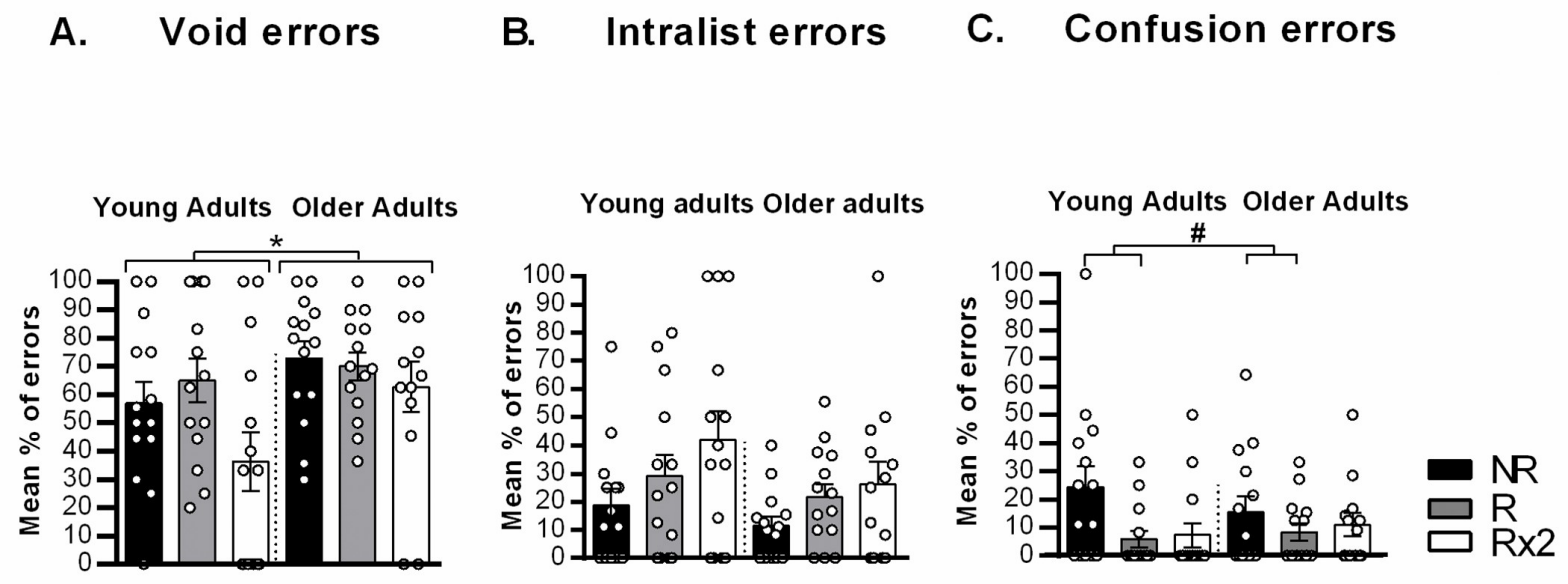
Mean percentage of errors at testing session for young and older adults. (A) Void type errors. (B) Intralist type errors. (C) Confusion type errors. *, p<0.05; #, p = 0.060.

Furthermore, we analyzed the percentage of type of errors performed at testing (Fig 3). Older adults showed significantly more percentage of void type errors than young adults (Fig 3A, F_age_(1,77) = 6.154 p = 0.015), but no differences were found neither between type of reminder (F_reminder_(2,77) = 3.059 p = 0.053) nor significant interaction (F_agexreminder_(2,77) = 0.936 p = 0.397). Moreover, no significant differences between type of reminder were found for the percentage of intralist type of errors (Fig 3B, F_reminder_ (2,77) = 2.181 p = 0.120) neither between age (F_age_(1,77) = 1.270 p = 0.263) and there was no significant interaction (F_agexreminder_(2,77) = 0.152 p = 0.859). However, there was significant differences between type of reminder for the percentage of confusion errors (Fig 3C, F_reminder_ (2,77) = 3.133 p = 0.05, but Bonferroni post-hoc tests revealed a trend for significant differences between NR vs. R (p_NR-R_ = 0.060 p_NR-Rx2_ = 0.117, p_R-Rx2_ = 0.958). There was no differences between age (F_age_(1,77) = 0.121 p = 0.729) neither significant interaction (F_agexreminder_(2,77) = 1.277 p = 0.285).

We further found no significant correlations between the level of ACE, IFS, age and memory change for NR, R and Rx2 groups for older adults (all ps>0.15 0.21<r<0.77). However, we found a significant trend for years of education and memory change for the Rx2 group (S1 Fig, r = 0.51 p = 0.06) but not for the NR and R groups (r = 0.22 p = 0.43, r = 0.28 p = 0.33, respectively).

Regarding the fluency task, there were no significant difference between groups nor sessions (Table 3, all ps>0.21), neither for any of the screening tests performed.

**Table 3:** Verbal fluency task.

	Training session		Testing session	
Groups	Young adults	Older adults	Young adults	Older adults
**NR**	13.1±1.0	12.6±1.5	11.9±0.9	11.1±1.2
**R**	14.8±1.2	11.7±0.8	13.9±1.1	11.5±1.1
**Rx2**	14.1±0.5	11.7±0.9	14.6±1.1	11.4±0.8
**p- value**	0.41	0.80	0.18	0.96

Mean number of responses in fluency task ± SEM at training and testing session.

NR stands for No reminder group, R stands for one round of reactivation group, Rx2 stands for two rounds of reactivation group.

It is important to highlight that for older adults the Rx2 group was significantly older than the NR group (Table 1, F(2,38) = 3.726 p = 0.03; Bonferroni post-hoc tests: p_Rx2-NR_ = 0.04 p_Rx2-R_ = 0.14 p_R-NR_ = 1.00). This could have negatively affected our results because it is well known that aging is negatively related with the ability to acquire, consolidate and recall new information [20, 35, 36], however, we found less memory decay for this group. Furthermore, there were no significant differences between groups at any demographic characteristics nor neuropsychological tests (Table 1, S2 Table).

## Discussion

Normal aging is accompanied by deficits in acquisition, consolidation and retrieval processes [21, 22]. It has been recently suggested that reconsolidation is affected as well [25–27]. In the present study, we showed that older adults had worse performance at training than young adults, revealing that encoding new information was affected in elderly as it has been previously demonstrated [20, 34, 37]. In the same line, regarding the type of errors distribution, older adults had a higher percentage of void type than young adults, independently the type of reminder presented. That is, older participants tended to give no answer when they were confronted to the retrieval cue, probably due to deficits in recall caused by aging [35, 36]. Finally, contrary to our hypothesis, memory reactivation process was not affected by aging since they showed memory strengthening after repeated reactivations to the same extent than young participants.

On one hand, the reminder treatment had the same effect independent on the participants age (no significant reminder x age interaction) showing that the double reactivation was more effective in protecting memory against forgetting than simple or no reactivation treatment. This effect was observed for absolute as well as for normalized memory change (Fig 2 and Table 2). However, for the absolute memory change young and older participants showed the same values meanwhile for the normalized one, the older adults showed a higher memory decay. That is, young and older adults lost the same mean amount of items between training and testing session but as the older adults reached a lower level of training the effect of forgetting, according to what they actually learned was more pronounced in this group.

Taking into account Jones et al. [25] and Sandrini et al. [26] studies, we can consider two main differences between their results and ours. One of them is memory reactivation. They used the spatial context for reactivating the memory trace whereas we used specific sounds and syllables to reactivate the stored information. We suggest that the sound plus the syllable could act as a stronger cue that facilitates memory reactivation than the context alone allowing memory labilization/reconsolidation. The other main difference is the type of retrieval, free recall against cue recall, respectively. It has been previously shown that aging affects more free recall than cued recall or recognition [35, 36]. Thus, deficits in free recall at aging in those studies could be hindering the reactivation effect.

However, it is important to highlight that in Sandrini et al. [26] study there is the possibility that the memory was reactivated by a spatial context. Unlike young adults [29], being in the same hospital might have been more salient to older adults than the distinction between the two experimental rooms, and the no reminder group would have been reminded of the learning session (Day 1) and performed the same as the reminder group. It has also been shown that older adults have a poorer memory of the source and also problems regarding the process of binding memories. So contextual cues may be encoded, but may not be properly bound to the specific episode [26, 38, 39]. More studies should be done comparing reminder effectiveness in triggering reconsolidation process in elderly.

Furthermore, St. Jacques et al. [27] observed memory reconsolidation, however they suggest an impact of aging in the process. The main difference to our results is that they use two different tour information (original and alternate one). In our study we use only one list of sound-word associations. Thus, as it was previously suggested, contextualization is impaired in old subjects [25, 26] thus, the lure information given after reactivation could be interfering at retrieval due to deficits in encoding the context or to the increase in source monitoring errors [40–43]. In the same line of our results, a recent study of Howe et al. [44] examined aging effects in episodic memory reconsolidation by reactivating memories in young and healthy older adults. On day 1, participants learned, to a strict criterion, eight pairs of novel images associated with unusual names On day 2, they received half of the images (term only) and were asked to silently remember their names (term). After that, they had to learn new eight associations. On day 8, they performed a recognition test formed by the eight pairs presented on day 1, the eight pairs of day 2 and sixteen unseen pairs. They had to indicate whether the pairing was old or new.

Consistent with our results, they observed that there were no differences at testing session between young and older adults at day 8. However, unlike previous studies, they found that there was no main effect of reactivation in young adults neither. We consider that this lack of reactivation effect could be possible due to the type of reminder used at day 2. It has been proposed that incomplete reminders, formed by part of the learned association are more effective in triggering memory labilization reconsolidation than complete ones [15, 45]. The incomplete reminders induce an error in prediction (a mismatch between expectation and reality) that triggers memory labilization allowing the subsequent memory modification. Thus, it can be argued that if during reactivation session participants silently remembered the associated names, the expectation could be accomplished, thus no error in prediction was detected and labilization was omitted.

Regarding strengthening effect induced by repeated reactivations, Forcato et al. [18] showed that in young adults the presentation of one or two reminders increased memory persistence on day 7 to an equal extent [19]. On the contrary, here we observed that only when two rounds of reminders were presented, memory was strengthened on day 7 in young as well as in older adults. The main differences between Forcato’s and the present study are 1) The type of material learned, that is, one list of nonsense syllables against sounds and known words associations; 2) The reminder session, one cue-syllable that labilizes the whole list against fifteen sound-first syllables of each association; 3) The variable used to measure memory reconsolidation, total number of errors at testing against memory change (correct responses at testing minus correct responses at training).

Recently, Bavassi et al. [46] performed a three-day experiment to characterize the post-treatment effect of a memory that went through different processes: reconsolidation, retraining or no treatment. On day 1, participants learned 36 image-word associations. On day 3, each participant received one round of reactivation: incomplete reminders for 1/3 of the associations, complete reminders for other 1/3 or no reminder for the last 1/3 of the associations. On day 5, subjects were tested. They observed that memory of the reactivated items was better than the no reactivation condition. Thus, the differences with our results, showing that one round of reactivation is not enough to increase memory persistence on day 7 could be due to the reminder session. That is, in Bavassi’s study each participant received each reminder condition, while in our study participants received only one reminder condition. It is interesting to ask whether the context in which the reminder is presenting could alter the error in prediction. That is, the complete reminders could be increasing the expectation for the incomplete reminders generating a greater error in prediction modifying the destiny of the memory.

We further found a trend for positive correlation between years of education and memory change for the Rx2 group, in older adults. Education is one known factor that increases the cognitive reserve throughout life [47]. Cognitive reserve is a hypothetical construct that provides an explanation for the unequal susceptibility to age—related brain changes between different people, whence some subjects could hold out these changes maintaining their cognitive functioning [48]. Thus, we suggest that the strengthening effect induced by the double reactivation could be reveal to a greater extent in a population with lower cognitive reserve where susceptibility to memory deficits is higher.

In line with previous reports [20, 36, 49], our results showed that encoding and retrieval are the more affected processes in elderly, thus, effective strategies are needed to reduce the natural memory loss. Taking under consideration that strengthening produced by repeated cued reactivations seems not to be affected in elderly, reconsolidation process could be an effective tool to reduce the impact of memory loss at aging. This is an important issue that should be taken into account for the design of new home-use tools to improve acquired information in old population.

## Supporting information

S1 Table Mean percentage of correct responses at day 7. (PDF)

S2 Table (XLSX)

S1 Fig Pearson Correlation between memory change and years of education in older adults. 95 degrees Confidence interval are shown.
(TIF)
