# Supplementary material for: Memory reconsolidation as a tool to endure encoding deficits in elderly
Source: PLoS One. 2020 Aug 7;15(8):e0237361. doi: 10.1371/journal.pone.0237361 (PMC7413497; doi:10.1371/journal.pone.0237361)
Supplement: S1 Table — (PDF) [file pone.0237361.s001.pdf]

S1 Table. Mean percentage of correct responses at day 7

Groups	Older adults	Young adults
**NR**	29.5±6.1	52.9±6.3
**R**	39.0±4.3	64.3±5.1
**Rx2**	56.4±5.8	82.4±3.3

Mean percentage of correct responses at day 7 (testing) ± SEM for young and older adults. NR, stands for the no reminder group; R, for one reactivation, and Rx2, for two reactivations.

We analyzed the testing session with two-way ANOVA with type of reminder as between subjects' factor with three levels (NR, R, Rx2) and age as between subjects factor with two levels (young, older), followed by Bonferroni Post-hoc test. Mean percentages are shown in table 2. There was no significant interaction between age and reminder condition ($F_{agexreminder}(2,77)=0.034$ $p=0.967$). Young adults performed better than older adults ($F_{age}(1,77)=33.617$ $p<0.001$) There was a significant difference between reminder conditions ($F_{reminder}(2,77)=14.623$ $p<0.001$). Specifically, the Rx2 group showed better performance than the NR and R groups ($p<0.001$ and $p=0.003$, respectively), but no differences were found between the NR and R groups ($p=0.144$).
