# Supplementary figures and images for: Memory reconsolidation as a tool to endure encoding deficits in elderly
Source: PLoS One. 2020 Aug 7;15(8):e0237361. doi: 10.1371/journal.pone.0237361 (PMC7413497; doi:10.1371/journal.pone.0237361)

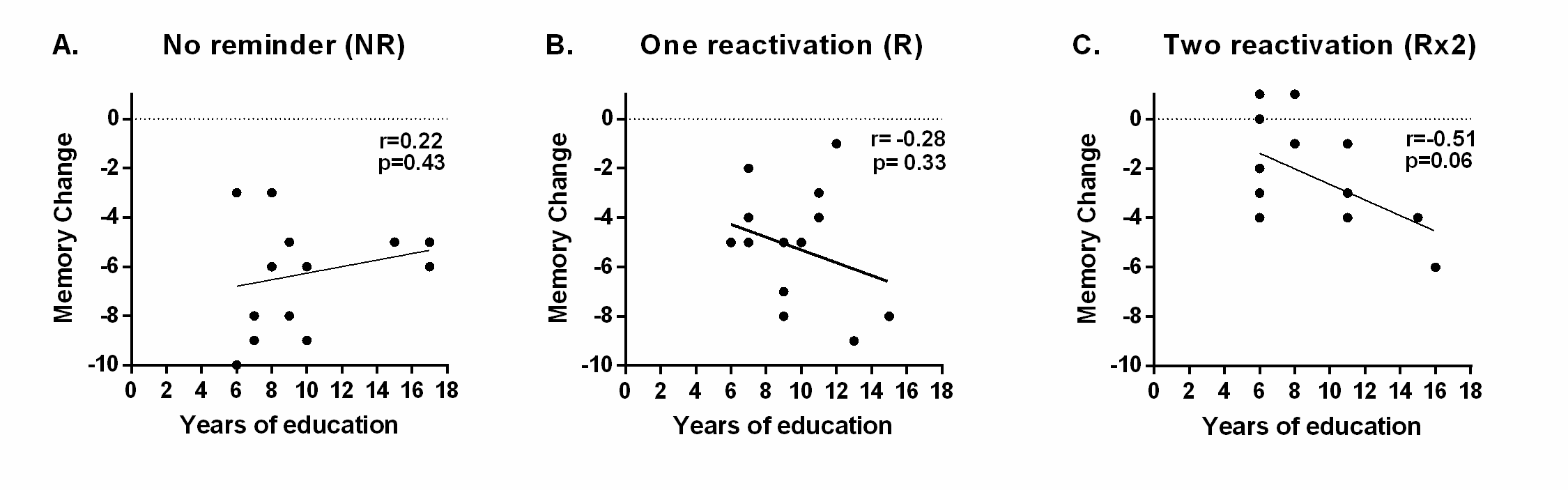

Supplement: S1 Fig — 95 degrees Confidence interval are shown. (TIF) [file pone.0237361.s003.tif]
